# The Burden of Neonatal Referrals on a Pediatric Cardiology Service: A Local Center Experience

**DOI:** 10.7759/cureus.47011

**Published:** 2023-10-14

**Authors:** Walaa S Al Maddallah, Yasser A Bhat, Abdulrahman Al Mesned, Abdullah Al Qwaee, Mohammad Ahmad Hassan, Ali Al Akhfash

**Affiliations:** 1 Pediatric Cardiology, Prince Sultan Cardiac Center, Buraidah, SAU; 2 Pediatric Department, Sohag Faculty of Medicine, Sohag University, Sohag, EGY

**Keywords:** neonate, cardiac referrals, neonatal intensive care unit (nicu), critical congenital heart disease, pediatric congenital heart disease

## Abstract

Background: Congenital heart disease (CHD) is a common occurrence in live births, with some exhibiting critical congenital heart disease; therefore, cardiology services should be available around the clock to ensure timely diagnosis and management. This study aims to describe the workload and the need for pediatric cardiac services in a maternity hospital for newborn referrals. Moreover, the study describes the indications for neonatal cardiology consultations.

Methods: The prospective cohort study was conducted over four months, from January to April 2022, in the Prince Sultan Cardiac Center Al Qassim region of Saudi Arabia. Prince Sultan Cardiac Center's pediatric cardiology department provides cardiac services to the Maternity and Children Hospital Buraidah Al Qassim. Out of the total 2,606 live births during the study period, the cardiology team evaluated 352 neonates. Neonates less than 30 days of age who were born in the maternity hospital were enrolled in the study. The outborn babies referred from other centers as suspected congenital heart disease for whom a cardiac evaluation was done were excluded. In addition, babies assessed in the emergency room and born elsewhere were excluded. Only new consultations have been considered, excluding follow-up consultations.

Statistical analysis: Data about patients' demographic, clinical and echocardiographic findings were recorded on Google Forms and converted to a Google spreadsheet. The Google spreadsheet's inbuilt statistical software was used for analysis. Categorical data were presented as percentages, and numerical data as median and range.

Results: The cardiology team evaluated 352 neonates from 2,606 live births over four months, accounting for 13.5 per 100 live births. The median weight was 2.8 kilograms, with a 0.5-4.3 kilogram range. Males comprised 187 (53%), and females comprised 165 (47%). Moreover, full-term, preterm, and post-term accounted for 236 (67%), 113 (32%), and 3 (0.8%) of patients, respectively. The common indications for neonatal cardiac referral were respiratory distress 60 (17%), infants born to diabetic mothers 50 (14%), abnormal fetal echocardiogram 49 (13.9%), family history of abortion or neonatal death 31 (8.8%), and congenital anomalies 30 (8.5%). Systolic murmur was the commonest clinical finding that prompted cardiology referrals 82 (23.2%), followed by desaturation 38 (10.7%) and dysmorphic features 31 (8.8%). Among the congenital cardiac defects, an isolated atrial septal defect (ASD) was seen in 66 (18.5%), isolated patent ductus arteriosus in 50 (14.2%), and ventricular septal defect in 21 (5.9%). Moreover, 13 (4.4%) lesions were critical CHDs. Finally, 27 (7.6%) had a diagnosis of pulmonary hypertension.

Conclusion: Knowing the burden of neonatal cardiac assessment on pediatric cardiology services in any maternity center may help the healthcare authorities to allocate resources and optimize the delivery of cardiac services among the neonatal population. Properly allocating pediatric cardiologists to the needed centers may optimize neonatal cardiac services. Moreover, it may decide on the number of pediatric cardiologists that need to be trained each year to meet the requirements of neonatal cardiac services.

## Introduction

Congenital heart disease (CHD) is reported in seven to nine live births in 1000 cases, with approximately 25% being a critical CHD, defined as a congenital heart condition requiring surgery/intervention or leading to death within the month following birth [1]. An estimated 261247 deaths worldwide were caused by CHD in 2017, with 69% occurring in infants under one year [2]. Severe CHD is a serious cardiovascular defect that can cause significant neonatal morbidity and mortality. Clinical presentations of critical CHD are shock, cyanosis, or respiratory distress, which can mimic other neonatal conditions [3]. Therefore, cardiology services should be available around the clock to ensure timely diagnosis and management. The lack of on-site pediatric cardiology services at many neonatal centers has led to the widespread use of neonatologists performing echocardiography under the supervision of pediatric cardiologists [4,5]. Additionally, pediatricians have a significantly different approach to evaluating and managing children with suspected CHD than pediatric cardiologists [6].

Moreover, the introduction of critical CHD screening for the early detection of critical CHD increased the need for comprehensive echocardiography [7]. This study aims to describe the workload and the need for pediatric cardiac services in a maternity hospital for newborn referrals. Moreover, the study describes the indications for neonatal cardiology consultations.

## Materials and methods

The prospective cohort study was conducted over four months, from January to April 2022, in the Prince Sultan Cardiac Center Al Qassim region of Saudi Arabia. Prince Sultan Cardiac Center's pediatric cardiology department provides cardiac services to the Maternity and Children Hospital Buraidah Al Qassim. The local Institutional Review Board approved the study (No. 21-1021). The Maternity and Children Hospital houses an 80-bed neonatal intensive care unit. Out of the total 2,606 live births during the study period, the cardiology team evaluated 352 neonates. Neonates less than 30 days of age who were born in the maternity hospital were enrolled in the study. The outborn babies referred from other centers as suspected CHD for whom a cardiac evaluation was done were excluded. 

In addition, babies assessed in the emergency room and born elsewhere were excluded. Furthermore, infants more than 30 days of age and children referred for cardiology evaluation from different pediatric subspecialties were excluded.

Exclusion of neonates evaluated in the cardiology outpatient department during the study period was also made. Only new consultations have been considered, excluding follow-up consultations, which may be fairly common in a neonatal intensive care unit. The secundum type of atrial septal defect (ASD) was defined as direct communication between the two atria at the fossa ovalis. At the same time, the patent foramen ovale was described as a channel with a septum primum flap on the left atrial side and the limbus of the septum secundum on the right atrial side [8].

A cardiology fellow is the first responder to neonatal referrals who evaluates patients and discusses them with a concerned consultant pediatric cardiologist. A consultant pediatric cardiologist gives the final opinion, and when required, patients are discussed in cardiology departmental meetings in the presence of cardiologists and cardiac surgeons.

Statistical analysis

Data about patients' demographic, clinical and echocardiographic findings were recorded on Google Forms and converted to a Google spreadsheet. The Google spreadsheet's inbuilt statistical software was used for analysis. Categorical data were presented as percentages, and numerical data as median and range.

## Results

The cardiology team evaluated 352 neonates from 2,606 live births over four months, accounting for 13.5 per 100 live births. The median weight was 2.8 kilograms, with a 0.5-4.3 kilogram range. Males comprised 187 (53%), and females comprised 165 (47%). Table 1 shows the number of cardiology referrals by gestational age.

**Table 1:** Cardiology referrals according to the gestational age. N: number; %: percent.

Gestational age (weeks)	N (%)
24–27.6	23 (6.5)
28–31.6	28 (7.9)
32–35.6	51 (14.4)
36–36.6	25 (7.1)
37–41.6	224 (63.6)
≥42	1 (0.2)
Total	352 (100)

Table 2 shows the number of neonatal cardiac referral indications.

**Table 2:** Main indication for a cardiology referral. N: number; %: percent.

Predominant reason for consultation	N (%)
Arrhythmia	1 (0.2)
Congenital anomalies	30 (8.5)
Diaphragmatic hernia	1 (0.2)
Convulsions	1 (0.2)
Cyanosis	7 (1.9)
Desaturation	1 (0.2)
Down syndrome	14 (3.9)
Dysmorphic features	1 (0.2)
Failed to wean from ventilation	1 (0.2)
Family history of abortion or neonatal death	31 (8.8)
Family history of congenital heart disease	5 (1.4)
Follow-up evaluations of fetal diagnosis	49 (13.9)
Heart murmur	36 (8.8)
Intra cytoplasmic sperm insemination	1 (0.2)
In vitro fertilization	1 (0.2)
Maternal diabetes	50 (14.2)
Maternal lupus	4 (1.1)
Maternal medications	2 (0.5)
Failed neonatal cardiac screening	4 (1.1)
To look for patent ductus arteriosus	15 (4.2)
Suspicion of persistent pulmonary hypertension of newborn	6 (1.7)
Prematurity	22 (6.2)
Renal anomalies	1 (0.2)
Respiratory distress	61 (17.3)
Wide pulse pressure	1 (0.2)
Total	352 (100)

Moreover, the clinical presentation of the referred neonates is presented in Table 3.

**Table 3:** Principal clinical features of the study population. N: number; %: percent.

Principal clinical features	N (%)
Normal	193 (54.8)
Desaturation	38 (10.7)
Dysmorphic	31 (8.8)
Sepsis	5 (1.1)
Systolic murmur	82 (23.2)
Weak femoral pulses	3 (0.8)
Total	352 (100)

Of the 352 referred neonates, 241 (68.4%) had an abnormal echocardiogram. Most infants had non-critical CHD, with only 13 (4.4%) of the 295 lesions being critical. The echocardiographic abnormalities are depicted in Table 4.

**Table 4:** Describes the predominant echocardiographic abnormalities of the referred neonates. N: number of lesions; %: percent.

Predominant lesion	N (%)
Aortic stenosis	2 (0.6)
Isolated atrial septal defect secundum type	75 (25.4)
Atrial septal defect secundum type associated with other congenital heart diseases	22 (7.4)
Atrioventricular septal defect	1 (0.3)
Isolated bicuspid aortic valve	3 (1.0)
Coarctation of aorta	4 (1.3)
D-transposition of great arteries with intact interventricular septum	1 (0.3)
Dextrocardia, corrected transposition, pulmonary atresia	1 (0.3)
Hypertrophic cardiomyopathy	1 (0.3)
Hypoplastic left heart syndrome	4 (1.3)
Severe mitral regurgitation	1 (0.3)
Partial anomalous pulmonary venous drainage	1 (0.3)
Isolated patent ductus arteriosus	50 (16.9)
Patent ductus arteriosus associated with other congenital heart diseases	96 (32.5)
Patent foramen ovale	24 (8.1)
Pulmonary hypertension	27 (9.1)
Pulmonary stenosis	2 (0.6)
Septal hypertrophy	7 (2.3)
Tricuspid atresia type II	1 (0.3)
Ventricular septal defect	21 (7.1)
Total	295 (100)

## Discussion

In the study, 13.5 newborns were referred for a cardiac assessment per 100 live births. The number of neonates who require cardiac evaluation at our center is significant. Based on the study, it is possible to estimate the need for cardiac service according to the number of live births.

The common indications for a cardiology referral were respiratory distress 60 (17%), infants born to diabetic mothers 50 (14%), follow-up evaluations of fetal cardiology 49 (13.9%), family history of abortion or neonatal death 31 (8.8%), and congenital anomalies 30 (8.5%). Systolic murmur was the commonest clinical finding that prompted cardiology referrals 82 (23.2%), followed by desaturation 38 (10.7%) and dysmorphic features 31 (8.8%). Geggel [9] found that systolic murmur was the most common reason for referral in newborns. Syndromic features and cyanosis were the next most common reasons. Moreover, fetal cardiology follow-up evaluations represented 14.1% of neonatal cardiac evaluations, almost similar to our study results. Khushu et al. [10] reported that almost 20% of the pediatric patients referred for cardiac evaluation due to asymptomatic murmurs have CHD. Moreover, a recent systematic review reported that 37% of newborns with asymptomatic, non-syndromic cardiac murmurs were diagnosed with echocardiography as having CHD. Therefore, the current practice of referring all newborns with murmurs for cardiac evaluation by echocardiography could be helpful in the earlier detection of CHD, thereby improving clinical outcomes [11].

Isolated atrial septal defect secundum type and patent ductus arteriosus were the most common cardiac lesions diagnosed, and patent ductus arteriosus was the most common defect associated with other congenital heart diseases (Table 4). This CHD distribution pattern among neonates reported in our study was similar to other previously published data [12]. CHD can be categorized into critical and non-critical lesions. Critical CHD includes ductal-dependent lesions for systemic or pulmonary blood flow like coarctation of the aorta, hypoplastic left heart syndrome, critical aortic stenosis, pulmonary atresia, and tricuspid atresia. The preoperative mortality rate of critical CHD reaches 5.4%; hence, these newborns' early diagnosis and management are crucial in this population [13,14]. Non-critical CHD includes ASD, ventricular septal defects, and bicuspid aortic valves [15]. In this study, 13 (4.4%) lesions were critical CHDs like dextro-transposition of great arteries, critical aortic stenosis, tricuspid atresia, hypoplastic left heart syndrome, and coarctation of the aorta. Minocha et al. [16] presented a study of 777 cardiology neonatal referrals, three of which had critical CHD.

The study emphasizes the burden of neonatal cardiac referrals in relation to the number of live births in a tertiary maternity center. It can give a good understanding of how to utilize and direct existing cardiac services, such as the evaluation of neonatal cardiac consultations.

Limitations

Only the new consultation was considered a referral. Repeated consultations for the same patient who had a previous cardiac evaluation were not taken as a referral. It would underestimate the burden of cardiac evaluations on pediatric cardiac services.

## Conclusions

Knowing the burden of neonatal cardiac assessment on pediatric cardiology services in any maternity center may help the healthcare authorities to allocate resources and optimize the delivery of cardiac services among the neonatal population. Properly allocating pediatric cardiologists to the needed centers may optimize neonatal cardiac services. Moreover, it may decide on the number of pediatric cardiologists that need to be trained each year to meet the requirements of neonatal cardiac services.
